# Attitude of Marlborough, Pewsey, and Swindon Doctors

**Published:** 1913-01-11

**Authors:** R. P. Beatty, H. B. Dismorr, A. E. L. Edwards, A. S. Gedge, T. R. Harvey, T. H. Haydon, J. C. Maclean, W. B. Maurice, G. K. Maurice, E. W. Rayment

**Affiliations:** Swindon.; Wroughton.; Upavon.; Pewsey.; Avebury.; Marlborough.; Swindon.; Marlborough.; Marlborough.; Pewsey.


### Attitude of Marlborough, Pewsey, and Swindon Doctors.

We take the following from the *Marlborough Times* of January 3, 1913:—

The attitude of medical men in Marlborough, Pewsey, Swindon, and district is shown by the following official communication:—

"We, the undersigned, desire to inform all whom it may concern that we have declined to accept service under the Insurance Act, in consequence of a pledge given to the British Medical Association, which we consider to be binding upon all honourable men. The pledge is as follows:—

I, the undersigned, hereby undertake that in the event of the National Insurance Bill becoming law, I will not enter into any agreement for giving medical attendance and treatment to persons insured under the Bill, excepting such as shall be satisfactory to the medical profession, and in accordance with the declared policy of the British Medical Association; and that I will enter into such agreement only through a Local Medical Committee representative of the Medical profession in the district in which I practise, and will not enter into any individual or separate agreement with any approved society or other body for the treatment of such persons.

"We have taken this step after the most careful consideration, and believe that we shall be upheld in this neighbourhood in the action we have taken. We should, however, regret that inconvenience should be caused to those who have been our patients. We are therefore ready to give our services free of charge to insured persons in our districts, until we give notice of other arrangements.

"We should be glad to meet representatives of those concerned in conference, if it is so desired.

R. P. Beatty, Swindon.
H. B. Dismorr, Wroughton.
A. E. L. Edwards, Upavon.
A. S. Gedge, Pewsey.
T. R. Harvey, Avebury.
T. H. Haydon, Marlborough.
J. C. Maclean, Swindon.
W. B. Maurice, Marlborough.
G. K. Maurice, Marlborough.
E. W. Rayment, Pewsey."

